# Dataset on the effect of soaking Kale (Brassica Oleraceae L. var. acephala DC.) seeds in solution based on amorphous silicon dioxide on the bioactive components and physiological growth parameters

**DOI:** 10.1016/j.dib.2022.107789

**Published:** 2022-01-05

**Authors:** Ali J. Othman, Ludmila G. Eliseeva, Polina G. Molodkina, Nazirya A. Ibragimova, Fatima M. Duksi

**Affiliations:** a Department of Commodity Research and Commodity Expertise, Plekhanov Russian University of Economics, Moscow, Russia; b Department of Natural Regenerated Resources and Ecology, Faculty of Agriculture, University of Aleppo, Syria

**Keywords:** Kale (Brassica Oleraceae L. var. acephala DC.), Microgreens, Capillary zone electrophoresis, Antioxidants capacity, Phenolic content, amorphous silicon dioxide

## Abstract

This dataset aims to evaluate the effect of different concentrations of amorphous silicon dioxide treatments on soilless-grown Kale (Brassica Oleraceae L. var. acephala DC.). Data were obtained from fresh and dry samples. Total antioxidants capacity, total phenolic content, ascorbic acid, chlorophyll-a, chlorophyll-b, total carotenoids concentrations, total nitrogen, growth parameters and germination percentage varied in response to the concentrations of the used treatments (amorphous silicon dioxide compounds). Spectrophotometry, coulometric analyzer, capillary zone electrophoreses were the principal involved methods. Data of antioxidant capacity, total phenolic, and ascorbic acid contents can provide significant physiological health benefits as a functional superfood. Total carotenoids, Chlorophyll a and b concentrations, total nitrogen content, dry matter content, plants height, fresh weights, and the percentage of seeds germination contribute to the understanding of biometric and physiological plants growth parameters that indicates the effectiveness of the used treatments.

## Specifications Table

**Table:** 

Subject	Food Science: Food Chemistry
Specific subject area	Food analysis, Horticultural Science, Hydroponics
Type of data	Table and raw data
How the data were acquired	Constant climate cabinet (Espec LHU-123, Germany) Coulometric analyzer (EXPERT-006, Econix-Expert, Russia) Spectrophotometer (Shimadzu UV 2401pc, Japan) Centrifuge (СМ-50, ELMI, Latvia) Centrifuge (OPN-8, NV-LAB, Russia) Capillary electrophoresis (Capel m-105, Lumex, Russia) Moisture analyzer (MX-50, A & D, Japan) Multi-detection microplate reader (Synergy HT, Bio-Tek, USA)
Data format	Raw and analyzed data
Description of data collection	The data on chlorophyll (a and b) and total carotenoid concentrations were obtained by spectrophotometry according to Lichtenthaler and Wellburn [1,2]. The data on total phenolic content were obtained by the colorimetric method of Folin–Ciocalteu [3] using gallic acid as a standard The data on total antioxidant capacity was obtained by coulometric analysis method using electrogenerated bromine radicals as described by Lapin A, [4]. The ascorbic acid contents data were obtained using capillary zone electrophoresis, the method described by Komarova and Othman [5,7]. The data on leaf total nitrogen were acquired according to the Kjeldahl method as described by Bremner [6].
Data source location	Russian economic University. G. V. Plekhanova, Stremyanny avenue, 36, Moscow, 115093, Russia Institution: Plekhanov Russian University of economics
Data accessibility	- Raw data (Supplementary material) - Data repository: https://doi.org/10.4121/17009009 - The mean and standard error of the experimental data is available in this article in Table 1.

## Value of the Data

• This dataset concerns the influence of soaking seeds with different concentrations of amorphous silicon dioxide preparation before sowing on the accumulation of bioactive components that form most of the nutritional value of kale (Brassica Oleraceae L. var. acephala DC.)
• This dataset might be useful for adjusting the dietary intake, especially when microgreens are considered a superfood that is rich in immunomodulators like polyphenolic compounds, vitamin C and K, and total antioxidants.
• These data are useful for microgreens growers by providing a methodology for enhancing the germination percentage of the crops.
• Data on chlorophyll (a and b) contents and accumulation of total nitrates provide additional information along with dry matter and microgreen's growth parameters in evaluating the physiological activities in the kale (Brassica Oleraceae L. var. acephala DC.) microgreens affected by soaking seeds with amorphous silicon dioxide solution.

## 1 Data Description

This dataset contains analyzed data derived from spectrophotometric, Kjeldahl, capillary zone electrophoresis, and coulometric analysis methods from fresh and dried samples of soilless-grown Kale microgreens in closed phytotron for 15 days. This dataset compares the influence of soaking Kale seeds in different concentrations of amorphous silicon dioxide on the content and accumulation of phytonutrients including ascorbic acid, total nitrogen content, total antioxidant capacity, total phenolic content, pigments concentrations, dry weight, and germination percentages in Kale microgreens.

## 2 Experimental Design, Materials and Methods

### 2.1 Microgreens cultivation and experimental treatments

One hundred Kale seeds were treated for 8 hours inside Petri dishes. In every petri dish Kale (Brassica Oleraceae L. var. acephala DC.) seeds were soaked in 15 ml of gradual concentrations of amorphous silicon dioxide (SD) solutions, where: SD1, SD2, SD3, SD4 refers to 50 mg.L^−1^, 100 mg.L^−1^, 200 mg.L^−1^, 333 mg.L^−1^, respectively. Petri dishes were placed inside a dark closed constant climate cabinet (Espec LHU-123, Germany) under a temperature of 22°C. Seeds were sown in growing substrates based on jute with dimensions of (17.5*47 cm) and placed in plastic pots and then placed in closed phytotron (ISR 0.1) conditions. The experiment was carried out on the 20th of August 2021 at Plekhanov Russian University of economics in the department of goods commodity and expertise of goods products, Moscow-Russian. Kale plants were drip-irrigated with mineral water Chernogolovka® (Aqualife, Moscow, Russia) of the following contents in mg.L^−1^: HCO3^−^ 122, Cl^−^ 11, SO₄²- 36, Mg^2+^, K^+^ 28, Na^+^ 22. The pH of mineral water ranged between 6.7-6.9, The electrical Conductivity recorded 1.9 mS.cm^−1^ and total dissolved solids 261 mg.L^−1^. Upon the third day, germinated seeds were exposed to a photoperiod of 18 h and 23/16 °C (day/night) temperature inside the phytotron. Photosynthetic photon flux density (PPFD) of 50 µmol m^−2^ s^−1^with red: green: blue = 1:1:1 light-emitting diodes (LEDs). The experiment comprised of randomized complete block design (RCBD), replicated six times for each treatment. Kale seeds were grown for 14 days after germination. Upon harvesting, the cotyledon stems of Kale microgreens were cut using sterile scissors.

### 2.2 Seeds germination percentage

Kale seeds were placed on dry Whatman filter papers in 28 cm diameter Petri dishes under 22°C temperature in a dark closed chamber. Exactly 100 seeds of Kale (Brassica Oleraceae L. var. acephala DC.). Then 15 mL of each treatment solution was slowly poured inside the Petri dish. Petri dish covers were placed on the bottom parts and left for 3 days. Germination percentages were performed by calculating the number of germinated seeds. 6 replicates were used for each treatment.

### 2.3 Dry weight

Dry weight contents were measured by placing exactly 1.00 g of whole Kale microgreens in a moisture analyzer (MX-50, A&D Co, Japan) until reaching constant weight under a constant temperature of 105 °C.

### 2.4 Total nitrogen content

Kale microgreens leaves were removed from their shoots, dried at a stable temperature of 70 °C for 72 h, and then macerated in a Wiley Mill before being used for the analysis. The total nitrogen content in the dried Kale microgreens samples was determined by the acid digestion method described by Bremner [6] conducted through 2 phases: firstly, by sample digestion to convert the nitrogen to ammonium followed by the determination of ammonium in the digest. Exactly 100 mg of macerated dry plant sample was added into Kjeldahl flask containing 1000 mg of selenium catalyst for accelerating the digestion process followed by 7 mL of 96% concentrated sulfuric acid and 5 mL of 32% of hydrogen peroxide. The mixture was stirred continuously for over 10 min until the digest became clear. Kjeldahl flask was cooled until 23 °C and then the content was placed into a 100 mL volumetric flask with distilled water and quantitatively made up to volume. A 5 mL aliquot of the digest was taken into a Markham distillation apparatus. Then 5 mL of 40% NaOH solution was added to the aliquot and the mixture was distilled. The distillate was collected in 5 mL of 2% H_3_BO_3_ solution. Three drops of a mixed indicator containing methyl red and methylene blue were added to the distillate in a 50 mL Erlenmeyer flask and then titrated with 0.01 M HCl acid solution. The percent of total nitrogen (N) was calculated from the titer value and then was converted to grams of nitrogen per kilogram of dry weight (DW).

### 2.5 Total antioxidants capacity

Galvanostatic coulometric analyzer (Econix-Expert, EXPERT-006, Russia) was used for total antioxidants capacity (AOC) in Kale microgreens samples using methods coulometric titration method with slight modifications [4]. Three grams of whole Kale microgreens were macerated and exactly 1.00 gm was transferred into the device's electrochemical cell that contains 50 ml of 0.1 M sulfuric acid (H2SO4) dissolved in 0.2 M Potassium Bromide (KBr). During mixing time, Bromine was generated under the application of a constant electrical current of 50 mA. During the reaction time, as a sample was injected, antioxidant substances quickly react with bromine radicals and the potential returns to its original value. The process of electro-titration was programmed by setting the potentials of the initial and end value of the indicator system on 40 mV and 200 mV respectively. The endpoint of the titration is fixed when the initial value of the indicator potential was reached. The device automatically filtered the bromine outflow, which was numerically equal to the number of antioxidants introduced in the aliquot. Total antioxidants capacity was determined as the Dihydroquercetin equivalent (mg DE/g) fresh weight from the calibration curve of Dihydroquercetin standard solutions.

### 2.6 Total chlorophyll and carotenoids

Pigments concentrations in Kale microgreens leaves were determined spectrophotometrically using the methods and equations of Lichtenthaler and Wellburn [1,2]. Exactly weighted 500 mg of fresh plant leaves sample was macerated with 5 mL of 96% ethanol, after 5 minutes of stirring another 5 mL were added then placed in a dark closed freezer under the temperature of −4°C for 30 min. The homogenized mixture was then transferred into 15 mL conical tubes and centrifuged for 8,000 rpm for 10 min at room temperature. Then 0.5 mL of the aqueous phase (supernatant) was poured into 4.5 mL of 96% ethanol and thoroughly mixed for 3 minutes. Samples were analyzed for total carotenoids, Chlorophyll-a, and Chlorophyll-b contents using a spectrophotometer (Shimadzu UV 2401pc UV-VIS, Japan).

### 2.7 Ascorbic acid determination

The content of ascorbic acid (Vitamin C) for the fresh samples was determined by capillary zone electrophoresis method [5,7] using (Kapel 105M, Lumex, Russia). 5 g of fresh sample was diluted to 100 cm^3^ and thoroughly mixed for 10 min in the dark then it was filtered and placed in Eppendorf tubes and centrifuged twice under 12000 rpm for 10 min to ensure the absence of any impurities. The supernatant was replaced into the device for analysis under the conditions of high positive voltage polarity. The internal diameter of the silica capillary is 50 µm and has a total length of 50 cm. Sodium tetraborate 10 mM was used as a buffer pH 9.2. Samples injected applying a pressure of 450 mbar.s^-1^, Voltage: +20 kV. Ascorbic acid was detected at a wavelength of A_254_ nm, at 24°C.

### 2.8 Total phenolic content

The total phenolic contents for the dry Kale samples were analyzed using the modified Folin–Ciocalteu colorimetric method [3]. A 50 mg of the dry sample was macerated for 5 min with 2 mL of ice-cold 95% methanol using ice-cold mortar and pestle. The homogenized mixture was then centrifuged at a rotation speed of 13,000 rpm for 10 min at a temperature of 23°C. Then, 1 mL of supernatant was mixed with 2.5 mL of 10% (w/v) Folin–Ciocalteu reagent. After 5 min, 2.0 mL of (20%) Na_2_CO_3_ was poured into the mixture and incubated at 45°C for 20 min with intermittent agitation. After 90 min of incubation in the dark at ambient temperature, the absorbance was read at A765 nm using a spectrophotometer (Shimadzu UV 2401pc UV-VIS, Japan). Total phenolic content was expressed as mg gallic acid equivalents (GAE) per gram of dry mass of Kale microgreens samples using a gallic acid calibration curve with regression of (R^2^=0.978).

### 2.9 Experimental data analysis

Raw Data analysis was subjected to one-way analysis of variance (ANOVA) using the IBM SPSS 25.0 version software (SPSS Inc., USA). Means separation for all treatments were done by Duncan's test at 5% level of significance for separation of treatment means within each measured parameter Table 1.

**Table 1:** Total nitrogen, TAC, ascorbic acid, total phenolic content, chlorophyll *a*, chlorophyll *b*, total chlorophyll, total carotenoids, dry weight, fresh weight, seeds germination, and height of Kale plants grown hydroponically in phytotron after seeds treatments with different concentrations of amorphous silicon dioxide.

Treatment	Total N (mg kg-1 FW)	TAC (mg DE/g FW)	Ascorbic acid (mg/100g)	Total phenolic content (mg GAE/g DW)	Total Carotenoids (mg/g) FW	Chl a (mg/g) FW	Chl-b (mg/g) FW	Total chlorophyll (mg/g) FW	FW (grams/100 plants)	Dry weight (%)	Plant Height, (cm)	Germination, (%)
Control	13.07 ± 0.46 cd	30.02 ± 1.13 d	37.49 ± 0.78 fg	147.17 ± 10.24 d	2.2 ± 0.04 d	4.79 ± 0.08 e	1.47 ± 0.08 cd	6.26 ± 0.07 d	5.18 ± 0.45 d	0.50 ± 0.01 d	5.44 ± 0.26 cd	72.42 ± 7.39 e
SD1	12.0 ± 0.5 d	53.34 ± 2.92 a	52.5 ± 08 d	279.34 ± 10.68 b	2.78 ± 0.20 b	7.17 ± 0.08 a	1.49 ± 0.12 cd	8.66 ± 0.09 ab	6.64 ± 0.16 bc	0.55 ± 0.0 b	8.31 ± 0.53 a	74.28 ± 3.81 d
SD2	15.45 ± 0.3 c	43.68 ± 0.47 c	41.5 ± 0.54 e	228.72 ± 13.46 c	2.35 ± 0.01 c	6.75 ± 0.03 b	1.72 ± 0.17 c	8.47 ± 0.16 b	6.49 ± 0.44 bc	0.52 ± 0.01 c	5.85 ± 0.51 c	82.89 ± 4.75 bc
SD3	29.1 ± 1.43 a	34.75 ± 0.43 cd	137.6 ± 3.7 a	137.58 ± 3.75 ef	2.13 ± 0.07 e	6.15 ± 0.11 c	2.69 ± 0.16 a	8.84 ± 0.05 ab	6.15 ± 0.11 c	0.60 ± 0.01 a	4.90 ± 0.22 e	82.69 ± 4.65 bc
SD4	29.17 ± 4.1 a	34.78 ± 2.12 cd	124.3 ± 8.5 b	124.31 ± 8.51 f	2.33 ± 0.11 c	6.88 ± 0.27 ab	2.15 ± 0.19 b	9.03 ± 0.20 a	6.88 ± 0.27 b	0.62 ± 0.01 a	4.25 ± 0.42 f	83.51 ± 6.48 b
N*	6	6	6	6	3	3	3	3	6	6	6	6

SD 1, SD2, SD3, SD4, Amorphous silicon dioxide 50 mg.L^−1^, 100 mg.L^−1^, 200 mg.L^−1^, 333 mg.L^−1^, respectively.

N*: number of repetitions. Different letters within each column indicate significant differences according to Duncan's test (P ≥ 0.05).

## Ethics Statement

There is no funding for the present effort. There is no conflict of interest. The data is available in public domain.

## CRediT authorship contribution statement

**Ali J. Othman:** Conceptualization, Visualization, Methodology, Software, Formal analysis, Writing – original draft. **Ludmila G. Eliseeva:** Conceptualization, Visualization, Resources, Formal analysis, Writing – review & editing. **Polina G. Molodkina:** Methodology, Software, Formal analysis. **Nazirya A. Ibragimova:** Methodology, Software, Formal analysis. **Fatima M. Duksi:** Writing – original draft, Formal analysis, Writing – review & editing.

## Declaration of Competing Interest

The authors declare that they have no known competing financial interests or personal relationships which have, or could be perceived to have, influenced the work reported in this article.

## Appendix Supplementary materials

Image, application 1
